# Isomorph Invariance of Higher-Order Structural Measures in Four Lennard–Jones Systems

**DOI:** 10.3390/molecules26061746

**Published:** 2021-03-20

**Authors:** Mahajabin Rahman, Benjamin M. G. D. Carter, Shibu Saw, Ian M. Douglass, Lorenzo Costigliola, Trond S. Ingebrigtsen, Thomas B. Schrøder, Ulf R. Pedersen, Jeppe C. Dyre

**Affiliations:** 1 Department of Physics, Emory University, Atlanta, GA 30322, USA; mahajabin.rahman@emory.edu; 2 Bristol Centre for Functional Nanomaterials, Tyndall Avenue, Bristol BS8 1TL, UK; benjamin.carter@bristol.ac.uk; 3 “Glass and Time”, IMFUFA, Department of Science and Environment, Roskilde University, P.O. Box 260, DK-4000 Roskilde, Denmark; shibus@ruc.dk (S.S.); ianmd@ruc.dk (I.M.D.); lorenzoc@ruc.dk (L.C.); trond@ruc.dk (T.S.I.); tbs@ruc.dk (T.B.S.); urp@ruc.dk (U.R.P.)

**Keywords:** Lennard-Jones system, Voronoi structures, Frank-Kasper bonds, icosahedral local order, bond-orientational order, density scaling, excess entropy, isomorph invariance, hidden scale invariance

## Abstract

In the condensed liquid phase, both single- and multicomponent Lennard–Jones (LJ) systems obey the “hidden-scale-invariance” symmetry to a good approximation. Defining an isomorph as a line of constant excess entropy in the thermodynamic phase diagram, the consequent approximate isomorph invariance of structure and dynamics in appropriate units is well documented. However, although all measures of the structure are predicted to be isomorph invariant, with few exceptions only the radial distribution function (RDF) has been investigated. This paper studies the variation along isomorphs of the nearest-neighbor geometry quantified by the occurrence of Voronoi structures, Frank–Kasper bonds, icosahedral local order, and bond-orientational order. Data are presented for the standard LJ system and for three binary LJ mixtures (Kob–Andersen, Wahnström, ${\mathrm{NiY}}_{2}$). We find that, while the nearest-neighbor geometry generally varies significantly throughout the phase diagram, good invariance is observed along the isomorphs. We conclude that higher-order structural correlations are no less isomorph invariant than is the RDF.

## 1. Introduction

While the structure of crystalline solids is well understood, the characterization of glass structure is much more challenging [1,2,3,4,5,6,7,8,9,10,11,12,13]. A glass is traditionally produced by cooling a liquid. At the glass transition temperature $T_{g}$ the liquid falls out of equilibrium and solidifies by basically freezing the atomic/molecular positions [14]. Thus, the structure of a glass is inherited from the liquid structure at $T_{g}$ (with occasional subtle exceptions [15]). This paper investigates liquid structure with an emphasis on glass-forming mixtures. The purpose is to illuminate how structure varies along a system’s isomorphs by using more detailed structure characterizations than the standard radial distribution function (RDF), which is a two-body isotropic correlation function.

If U(R) is the potential energy as a function of all *N* particle coordinates $R\equiv (r_{1},\;\ldots ,\;r_{N})$, $R_{a}$ and $R_{b}$ are two configurations at the same density, and λ is a uniform scaling parameter, hidden scale invariance is the following property [16,17,18,19]:

(1) $$U\left(R_{a}\right)<U\left(R_{b}\right)\;\;\Rightarrow \;\;U\left(\lambda R_{a}\right)<U\left(\lambda R_{b}\right)\;.$$

In words, this logical implication says that if configurations at one density are ordered according to their potential energy, the ordering is maintained if all configurations are scaled uniformly to a different density. Intuitively, one expects this to have consequences for how equilibrium structures change with density, but what are these consequences? The answer is that, whenever Equation (1) applies, structure and dynamics are invariant along the configurational adiabats in the thermodynamic phase diagram, the so-called isomorphs [16]. This result is exact if Equation (1) applies without exceptions; this is the case only when U(R) is an Euler homogeneous function, i.e., obeys $U\left(\lambda R\right)=\lambda^{-n}U\left(R\right)$ for some exponent *n*. In cases of relevance in experiments and simulations, hidden scale invariance applies only for the majority of the physically relevant configurations and for λ fairly close to unity. In such cases, structure and dynamics are not rigorously isomorph invariant. Simulations of several different atomic and molecular models have demonstrated, however, that structure and dynamics are still invariant along an isomorph to a good approximation [18,20,21,22,23,24]. In regard to the dynamics, this finding confirms Rosenfeld’s excess-entropy-scaling principle of 1977 [18,25]. In fact, it may be shown that if a system has curves in its phase diagram along which the atoms/molecules move about each other such that the same movie would be recorded at different state points (except for scaling of space and time), then state points on these curves have the same excess entropy and Equation (1) must apply [18].

The degree to which Equation (1) applies varies throughout the phase diagram. For instance, most systems do not exhibit hidden scale invariance near the critical point. We henceforth consider only systems and regions of the phase diagram for which Equation (1) applies to a good approximation. For any such system, an isomorph is defined as a line of constant excess entropy $S_{\mathrm{ex}}$ in the phase diagram ($S_{\mathrm{ex}}$ is the entropy minus that of an ideal gas at the same temperature and density [25,26,27], a quantity that is negative because any system is more ordered than an ideal gas). A convenient way of checking whether Equation (1) applies to a good approximation is to evaluate the virial potential-energy Pearson correlation coefficient *R* [28],

(2) $$R\;\equiv \;\frac{\langle \Delta U\Delta W\rangle }{\sqrt{\langle {\left(\Delta U\right)}^{2}\rangle \langle {\left(\Delta W\right)}^{2}\rangle }}\;.$$

Here *W* is the microscopic virial function, which for a three-dimensional pair-potential system is given [26] by $W\left(R\right)=\sum_{i<j}r_{ij}\cdot F_{ij}/3$ in which $r_{ij}$ is the position vector from particle *i* to particle *j* and $F_{ij}$ is the force on particle *j* from particle *i* [26], Δ denotes the quantity in question minus its state-point average, and the sharp brackets indicate NVT canonical-ensemble averages. A useful rule of thumb is that if R>0.9, the system obeys hidden scale invariance and has, consequently, good isomorphs [20]. In that case, the system is referred to as “strongly correlating” or “R-simple” [22,28]. The latter name makes it possible to distinguish this class from “simple liquids”, which are traditionally defined as systems of particles interacting via pair forces [27,29]. While many such systems have good isomorphs, some do not; on the other hand, molecular and other more complex liquids may well have strong virial potential-energy correlations and the consequent isomorphs [18,22]. Isomorph invariance of structure and dynamics implies that the phase diagram becomes essentially one-dimensional wherever hidden scale invariance applies. This provides a significant simplification for understanding and describing a given system.

Isomorph invariance of structure and dynamics applies only when these are given in “reduced” units [20,22]. If one considers *N* particles in volume *V* at temperature *T*, the (number) density is defined by ρ=N/V, and the units used for defining reduced quantities are the length $l_{0}\equiv \rho^{-1/3}$, the energy $e_{0}\equiv k_{B}T$, and the time $t_{0}\equiv \rho^{-1/3}\sqrt{m/k_{B}T}$ [20] in which *m* is the average particle mass. These are sometimes referred to as “macroscopic” units [25]; note that these units depend on the thermodynamic state point in question. Reduced quantities are generally marked by a tilde, for instance $\tilde{r}\equiv r/l_{0}=\rho^{1/3}r$ is the reduced version of the distance *r* between two particles.

Isomorph invariance of reduced-unit RDFs has been reported for several different liquid and crystalline systems [20,21,30,31,32,33,34,35,36]. This is investigated by plotting the RDF as a function of $\tilde{r}$ for different state points along an isomorph to see whether there is data collapse. A recent application showed how the invariance along isomorphs can be utilized to predict the structure of an R-simple liquid at an arbitrary state point from a single simulation [37].

The theory predicts isomorph invariance of any structural measure, not just the RDF. Only few studies have been carried out to check this prediction, however. Ingebrigtsen and Tanaka demonstrated good isomorph invariance of the bond-orientational order in polydisperse Lennard–Jones (LJ) systems of both size and energy dispersity [38,39]. On the other hand, an investigation in 2013 by Malins et al. [40] of the Kob–Andersen (KA) binary LJ system [41] reported significant isomorph variation of the number of 11A bicapped antiprism clusters. Malins et al. concluded that “these higher-order structural and dynamical correlations show very much larger deviations along the [KA] Lennard–Jones isomorphs than do two-body correlations. This result is at odds with the invariance of structure in reduced units predicted by the theory of isomorphs.” By a higher-order structural measure is meant a quantity, the calculation of which involves the relative positions of more than two particles. Such measures provide much more details of the nearest-neighbor geometry than the RDF does.

To investigate the generality of the finding of Ref. [40], there is a need for more data on higher-order structures of different R-simple systems. In this paper, we investigate different higher-order structural measures of four different LJ systems. The measures involve Voronoi structures, Frank–Kasper bonds, icosahedral local order, and bond-orientational order. Overall, we find a good isomorph invariance of these structural measures. The system studied are the standard single-component LJ system (Section 2.1), as well as the binary Kob–Andersen, Wahnström, and ${\mathrm{NiY}}_{2}$ LJ mixtures (Section 2.2). Computational details are provided separately in each section and Appendix A provides isomorph state-point details.

## 2. Results and Discussion

### 2.1. Standard Lennard–Jones System

The LJ pair potential v(r) is defined [42] by

(3) $$v\left(r\right)\;=\;4\varepsilon \left[{\left(\frac{r}{\sigma }\right)}^{-12}-{\left(\frac{r}{\sigma }\right)}^{-6}\right]\;.$$

The parameter ε sets the energy scale and σ sets the length scale. The LJ pair potential is strongly repulsive at short distances and diverges as r→0; it has a global minimum at $r=2^{1/6}\sigma$ at which $v(2^{1/6}\sigma )=-\varepsilon$. Simulations of LJ systems usually employ the so-called LJ units defined by ε and σ. In tests of isomorph theory it is important, however, to report quantities in the above-mentioned macroscopic unit system. Note that specifying the state point itself is not possible in reduced units because $\tilde{\rho }=\tilde{T}=1$ at all state points; thus state points are reported in LJ units.

This section reports results for the standard single-component LJ system. The focus is on how the relative fractions of different Voronoi structures vary along isomorphs. To put the findings into perspective, we also performed the same analysis along isochores, i.e., for constant-density state points.

**Figure 1:**
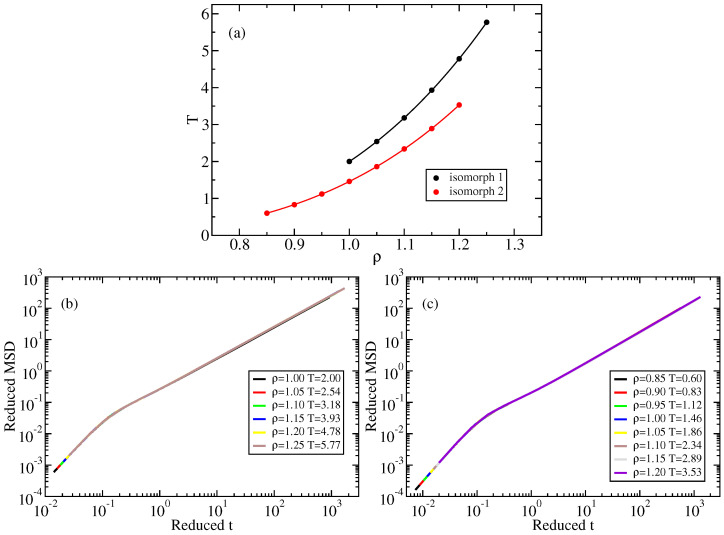
(**a**) The two LJ isomorphs studied. State points on the isomorphs were found by integrating Equation (4) numerically. (**b**) Mean-square displacement (MSD) for the state points of isomorph 1 plotted in reduced units, demonstrating good isomorph invariance of the dynamics. (**c**) The same for the state points of isomorph 2.

The simulations were carried out using the open-source Roskilde University Molecular Dynamics software (RUMD v3.5) that runs on graphics processing units [43] (http://rumd.org (accessed on 4 September 2020)). The LJ liquid was simulated by standard Nosé-Hoover NVT dynamics with a thermostat relaxation time of 0.2 for a system of N=8000 particles. A shifted-potential cutoff was employed at 2.5σ. We studied two isomorphs, one that is above the freezing line (“isomorph 1”, with reference state point (ρ,T)=(1.00,2.00)) and one that is slightly supercooled (“isomorph 2”, with reference state point (ρ,T)=(0.85,0.60)). Supplementing this, we also investigated the ρ=1.00 isochore that has state points overlapping with both isomorphs, as can be seen in Figure 1a that shows the two isomorphs in the thermodynamic phase diagram.

Isomorphs are defined as lines of constant excess entropy in the phase diagram of an R-simple system, i.e., a system with hidden scale invariance [16,20]. Two isomorphs were generated by integrating Equation (4) below, which defines the density-scaling exponent γ at a given state point [20]:

(4) $$\gamma \;\equiv \;{\left(\frac{\partial \ln T}{\partial \ln\rho }\right)}_{S_{\mathrm{ex}}}\;=\;\frac{\langle \Delta U\Delta W\rangle }{\langle {\left(\Delta U\right)}^{2}\rangle }\;.$$

The last equality is a statistical-mechanical identity, which allows for calculating γ from canonical-ensemble constant-volume (NVT) averages [20]. If for instance γ=3, Equation (4) implies that when the density is increased by 1%, the temperature should be increased by 3% in order to stay on the isomorph. To perform the integration accurately, we used the fourth-order Runge-Kutta algorithm for density changes of 5%.

To verify that the state points of Figure 1a are indeed on isomorphs, we check in Figure 1b,c that there is a collapse of the dynamics by plotting the mean-square displacement (MSD) as a function of time in reduced units. We note that the short-time (ballistic) collapse of the reduced MSD follows from the definition of reduced units and applies to any system at any state point, independent of hidden scale invariance. The long-time collapse, however, is not trivial, and demonstrates isomorph invariance of the dynamics.

The software Voro++ [44,45] was used to obtain data of the Voronoi construction around each particle. The output was analyzed by using the number of edges, faces, etc, as parameters to classify an environment. From this we calculated the fraction of particles with a given local environment. We focused on the four most common environments defined by the number of edges, vertices, and faces of the Voronoi polyhedron surrounding a particle. This characterization allows one to include nearly 90% of the local environments in the investigation.

**Figure 2:**
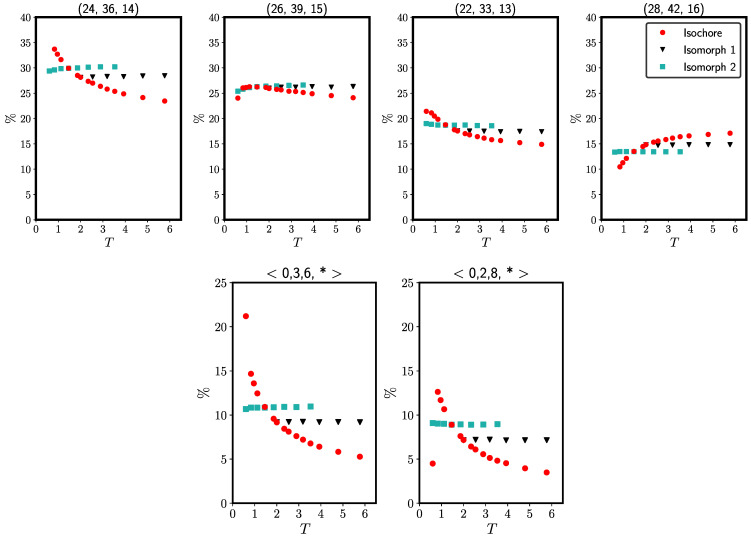
Voronoi-structure variation along the two isomorphs and along the ρ=1.00 isochore of the single-component LJ system. Top panels: Fraction of local environments as a function of temperature for four common occurrences of the number of edges, vertices, and faces (marked on top of each figure). There is some variation along the isomorphs, but it is much smaller than along the isochore. The deviations decrease with increasing temperature and the consequent increase of *R* (Appendix A). Bottom panels: Fraction of the two most common Voronoi polyhedra of the conventional indexing system. Again we find approximate isomorph invariance.

Figure 2 shows the temperature variation of Voronoi characterizations of local environments. In the upper figures, each subfigure represents a single local environment specified by three integers giving the number of edges, vertices, and faces, respectively, of the Voronoi polyhedron around a given particle.. The fraction of particles with this particular environment is shown on the y-axis as a function of the temperature. The two lower figures give the temperature variations of the standard Voronoi indices in the form $\langle n_{3},n_{4},n_{5},n_{6}\rangle$ where $n_{3}$ is the number of triangles, $n_{4}$ the number of rectangles, etc, of the Voronoi polyhedra. These figures show data for the occurrence of the two most abundant Voronoi structures; note that a star is a wildcard, thus a sum over several Voronoi structures is represented in the lower figures.. In both the upper and lower figures, the structures are almost invariant along the two isomorphs, but not along the ρ=1.00 isochore.

### 2.2. Binary Lennard–Jones Mixtures

The single-component LJ system easily crystallizes in the supercooled regime. A simple way to avoid this is to consider binary mixtures. This section gives results for higher-order structures of the Wahnström, Kob–Andersen, and ${\mathrm{NiY}}_{2}$ binary LJ mixtures.

#### 2.2.1. Wahnström Mixture

The Wahnström system is defined [46] by having an equimolar composition of two particles, A and B, that interact via LJ potentials with $\sigma_{AA}=1.0,\;\sigma_{AB}=1.1,\;\sigma_{BB}=1.2$, and $\varepsilon_{AA}=\varepsilon_{AB}=\varepsilon_{BB}=1.0$. The mass of particle B is twice that of particle A. For all three interactions a shifted-potential cutoff was used at $r_{cut}=2.5\;\sigma$ with the relevant σ. Like all systems of this study, the Wahnström mixture was simulated by Nosé-Hoover NVT dynamics in RUMD. The time step was $0.002\;\rho^{-1/3}T^{-1/2}$ (in LJ units) for the isomorph simulations and 0.005 for the isochore simulations. Systems were equilibrated for 5 million time steps at each state point before a data-collection run of 10 million time steps.

Isomorphs were identified using an approximate analytical formula that applies to a good approximation for any R-simple LJ system, both single- and multicomponent systems. According to this, the temperature variation T(ρ) as a function of the density along the isomorph through the reference state point $\left(\rho_{0},T_{0}\right)$ is given by [47,48,49].

(5) $$\frac{T(\rho )}{T_{0}}=\left(\frac{\gamma_{0}}{2}-1\right){\left(\frac{\rho }{\rho_{0}}\right)}^{4}-\left(\frac{\gamma_{0}}{2}-2\right){\left(\frac{\rho }{\rho_{0}}\right)}^{2}\;.$$

Here $\gamma_{0}$ is the density-scaling exponent at the reference state point, which is calculated from equilibrium canonical (NVT) fluctuations by means of Equation (4). The first term of Equation (5) derives from the repulsive $r^{-12}$ term of the LJ pair potential and the second term derives from the attractive $r^{-6}$ term. This method for tracing out an isomorph is convenient because it requires only a single simulation. We checked that the correct isomorphs are traced out by also integrating Equation (4) numerically.

**Figure 3:**
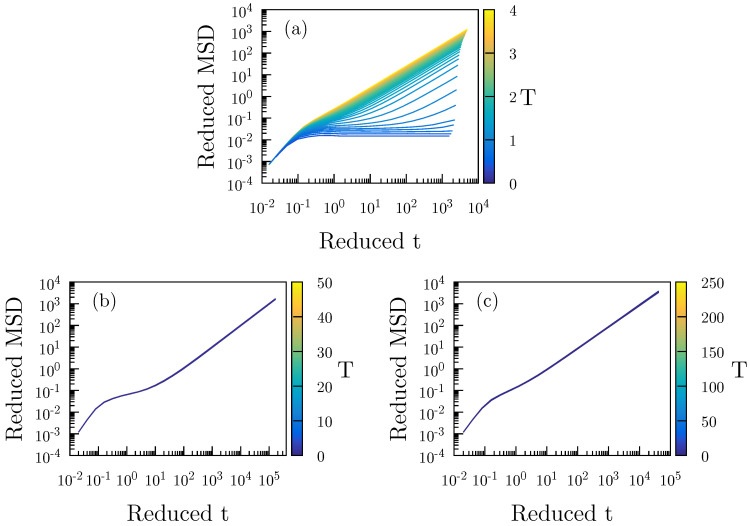
A particle mean-square displacement in reduced units of three different sets of simulations of the Wahnström binary LJ mixture [46]. (**a**) gives MSD data along the ρ=0.85 isochore (where density is given in AA particle units). (**b**) is for the isomorph with reference state point (ρ,T)=(0.85,1.2). (**c**) is for the isomorph with reference state point (ρ,T)=(0.85,2.0).

Figure 3 gives reduced-unit MSD data for the A particles as functions of time along an isochore (Figure 3a) and two isomorphs (Figure 3b,c). The MSD is isomorph invariant to a good approximation, while it varies significantly along the isochore.

We proceed to the investigation of higher-order structures. Voronoi structures were again calculated using the Voro++ library [45]. Frank–Kasper (FK) bonds [50] were determined using a “neighbor” cutoff at approximately the first minima in the RDF: 1.7 and 1.8 for AB and BB, respectively, after a uniform rescaling of the system to ρ=0.85. BB pairs with six A particles (and none other) in their common neighbor list were declared to be a FK bond [50].

**Figure 4:**
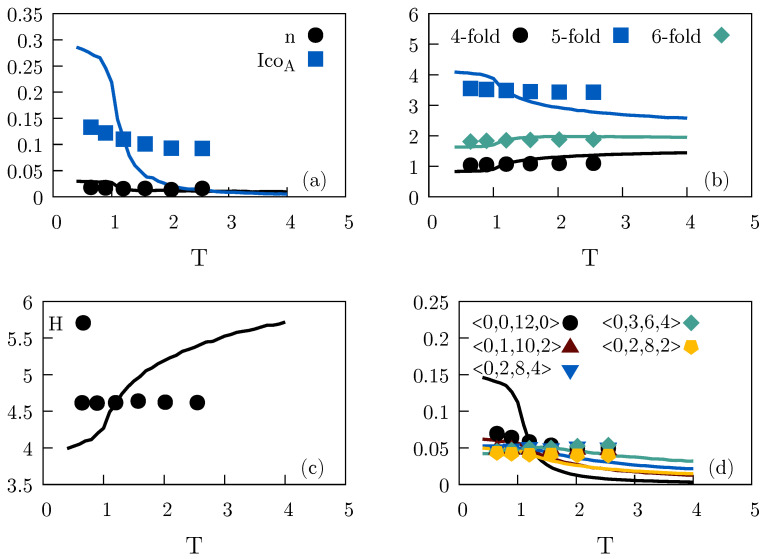
Different higher-order structure measures along an isomorph and along the isochore of Figure 3a, plotted as functions of the temperature for the Wahnström binary LJ mixture. The solid lines represent values along the ρ=0.85 isochore, while the points give values along the isomorph generated from the reference state point (ρ,T)=(0.85,1.2) (Figure 3b). (**a**) shows results for the occurrence of Frank–Kasper bonds (black, denoted by “*n*”) and small particles in icosahedral local order (blue, denoted by “${\mathrm{Ico}}_{A}$”). (**b**) shows the average number of four-, five-, and six-sided faces of the Voronoi polyhedra. (**c**) shows the Shannon entropy of the cell types of the Voronoi tessellation (Equation (6)). (**d**) shows the relative frequency of occurrence of the five most common Voronoi cell types. Overall, there is good isomorph invariance.

Figure 4 shows how different higher-order measures of the local structure vary along the reference-state-point (ρ,T)=(0.85,1.2) isomorph as a function of the temperature (symbols) and how they vary along the ρ=0.85 isochore (full curves). Figure 4a gives two measures of preferred local structures: the number of Frank–Kasper bonds [51,52] denoted by *n* (normalized per large particle) and the number of small particles in an icosahedral local order denoted by “${\mathrm{Ico}}_{A}$”. Both measures are found in the optimal crystal structure MgZn${}_{2}$, which is a Laves-type crystal, as well as in the supercooled liquid [52,53]. In Figure 4b we show the number of four-, five-, and six-sided faces in the Voronoi tessellation. In Figure 4c we report the Voronoi-structure Shannon entropy *H*, a standard quantity in information theory defined by

(6) $$H\;=\;-\sum p_{i}\ln p_{i}\;,$$

of the cell types of the Voronoi tessellation in which $p_{i}$ is the relative frequency of cell type *i*. *H* measures the diversity of cell types in the system, with larger values of *H* corresponding to a wider range of probable structures and H=0 corresponding to a unique structure like the crystal. *H* increases a lot with temperature along the isochore. This reflects the fact that the high-temperature liquid is more diverse than the cooler liquid [13]. On the other hand, if the system is compressed when temperature is increased to stay on the isomorph, *H* is virtually constant.

Finally, in Figure 4d we look at the five most common Voronoi structures [54,55] (identified at the (ρ,T)=(0.85,1.2) reference state point). None of these structures dominate, however, with the most common one being less than 8% likely along the isomorph. In Figure 4d the $\langle n_{3}$, $n_{4}$, $n_{5}$, $n_{6}\rangle$ notation is used in which $n_{i}$ is the number of faces with *i* sides of the Voronoi polyhedron. Note the prevalence of the 〈0,0,12,0〉, 〈0,1,10,2〉, and 〈0,2,8,2〉 structures; the first of these is the ideal dodecahedron corresponding to icosahedral local ordering, while the two others are perturbations of the dodecahedron. These five structures account for roughly a third of all structures.

Figure 4 shows that most local structures of the Wahnström system are close to invariant along the isomorph while, over the same temperature range, the structures vary considerably along the isochore. In a few cases, isomorph invariance breaks down at the lowest temperatures, in particular for the small particle icosahedral structures (Figure 4a) and the regular 〈0,0,12,0〉 Voronoi structures (Figure 4d.) This is a reminder that isomorph theory is only exact for unrealistic systems with an Euler homogeneous potential-energy function. Since the 〈0,0,12,0〉 Voronoi structure is that of the fcc crystal, another possibility is that the system is slowly crystallizing

**Figure 5:**
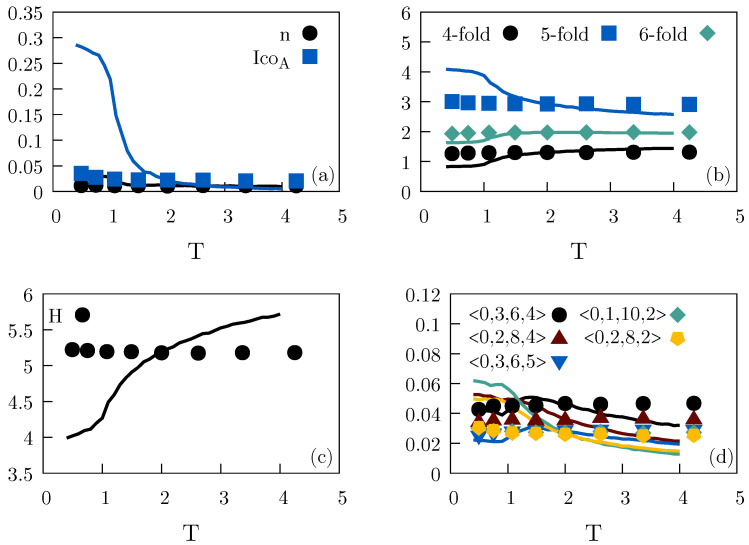
As in Figure 4, but for the isomorph with reference state point (ρ,T)=(0.85,2.0), which is above the freezing line. The isochore is given by ρ=0.85. The structures are isomorph invariant to a good approximation, but vary along the isochore. Note that compared to Figure 4 there are more low-probability Voronoi structures, with no dominant structural motif.

Figure 5 is similar to Figure 4, but for the isomorph generated from the reference state point (ρ,T)=(0.85,2.0). While many of the structural measures remain isomorph invariant, we note a minor variation of the icosahedral local ordering with temperature (blue points in (a)). Most likely, this influences several of the other structural measures.

#### 2.2.2. Kob–Andersen Mixture

The Kob–Andersen (KA) system is a 4:1 mixture of two particles, A and B, interacting via LJ potentials with $\sigma_{AA}=1.0,\;\sigma_{AB}=0.8,\;\sigma_{BB}=0.88$, $\varepsilon_{AA}=1.0$, $\varepsilon_{AB}=1.5$, and $\varepsilon_{BB}=0.5$ [41]. Note that the A particle is larger than the B particle, while the opposite is the case for the Wahnström system. The mass of particle B is equal to that of particle A. For all three interactions a shifted-potential cutoff was used at $r_{cut}=2.5\;\sigma$ with the relevant σ. The time step used in the simulations was $0.001\rho^{-1/3}T^{-1/2}$ and the NVT thermostat relaxation time was 0.2. The system was equilibrated for 5 million time steps at each state point before a production run of 500 million time steps. The simulations involved 4000 particles. Voronoi structures were calculated using the Voro++ library [45].

**Figure 6:**
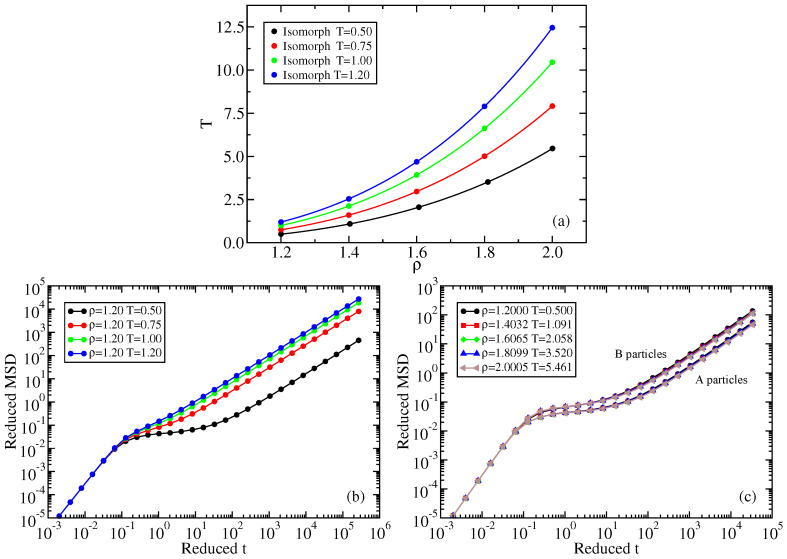
Kob–Andersen (KA) binary LJ system characteristics. (**a**) Four isomorphs. (**b**) Reduced A particle MSD at four state points of the ρ=1.20 isochore. (**c**) Reduced MSD along the lowest-temperature isomorph (reference state point (ρ,T)=(1.20,0.50)) for the A and B particles, respectively, confirming that the state points are isomorphic by collapsing the reduced-unit mean-square displacements as a function of the reduced time. Note that the B particles are considerably faster than the A particles.

Figure 6a shows four isomorphs, generated from reference state points of density 1.2 and temperatures 0.5, 0.75, 1.0, 1.2, respectively. The T=0.5 isomorph was generated by integrating Equation (4) numerically, while the remainder were identified by means of Equation (5) (Appendix A gives state-point information). Figure 6b shows the reduced A-particle MSD along the ρ=1.20 isochore at the temperatures of the four isomorph reference state points. Figure 6c shows both the A and B particle reduced-unit MSDs along the lowest-temperature isomorph. Despite the fact that the temperature variation along the isomorph is considerably larger than along the isochore, there is good isomorph invariance.

**Figure 7:**
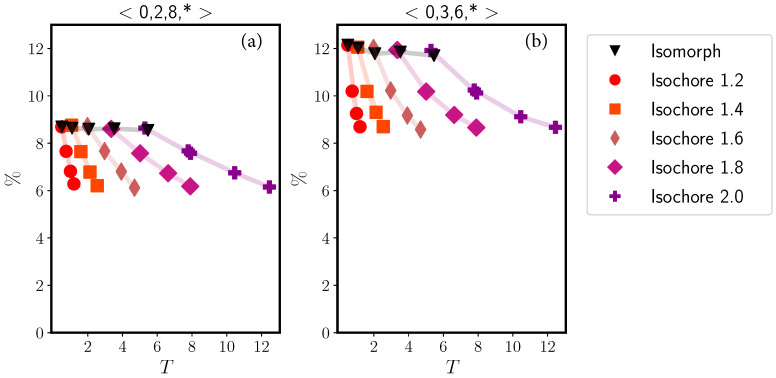
Two Voronoi structures of the KA system probed along the reference state point (ρ,T) = (1.20,0.50) isomorph and along several isochores, plotted as a function of the temperature. (**a**) shows results for the <0,2,8,∗> Voronoi structure around either an A or a B particle, and (**b**) shows analogous results for the <0,3,6,∗> Voronoi structure. While there is significant variation along the isochores, both structures are isomorph invariant to a good approximation.

In Figure 7 we study the variation along the isomorph with reference state point (ρ,T)=(1.20,0.50) and along several isochores of two classes of Voronoi structures around the B particles, those with <0,2,8,∗> Voronoi indices and those with <0,3,6,∗> indices where ∗ is a wildcard. There is little variation along the isomorph, while the structures become systematically less likely as temperature increases along the isochores. The latter is because at high temperatures, more structures contribute sizably to the statistics.

We next consider the time-autocorrelation function of the bond-orientational order parameter defined [56] by

(7) $$C_{6}\left(t\right)\equiv \langle \sum_{m}{\bar{Q}}_{6m}^{i}\left(t\right)\;{\bar{Q}}_{6m}^{i*}\left(0\right)\rangle \;,$$

in which ${\bar{Q}}_{6m}^{i}\left(t\right)$ is a coarse-grained Steinhardt bond-orientational-order parameter for particle *i* at time *t*:

(8) $${\bar{Q}}_{6m}^{i}\left(t\right)\equiv \frac{1}{{\tilde{N}}_{b}\left(i\right)}\sum_{k=1}^{{\tilde{N}}_{b}\left(i\right)}Q_{6m}^{k}\left(t\right)\;,$$

with

(9) $$Q_{6m}^{k}\left(t\right)\equiv \frac{1}{N_{b}\left(k\right)}\sum_{j=1}^{N_{b}\left(k\right)}Y_{6m}\left(r_{kj}\left(t\right)\right)\;.$$

Here $Y_{6m}$ is the spherical harmonic function of degree *l* = 6 and order m=−6, …, 6, $N_{b}\left(k\right)$ is the number of neighbors of particle *k*, and ${\tilde{N}}_{b}\left(i\right)$ is the number of neighbors of particle *i* including the particle *i* itself. “Nearest-neighbor particles” are defined using the Voronoi construction and identified with the Voro++ package [45].

**Figure 8:**
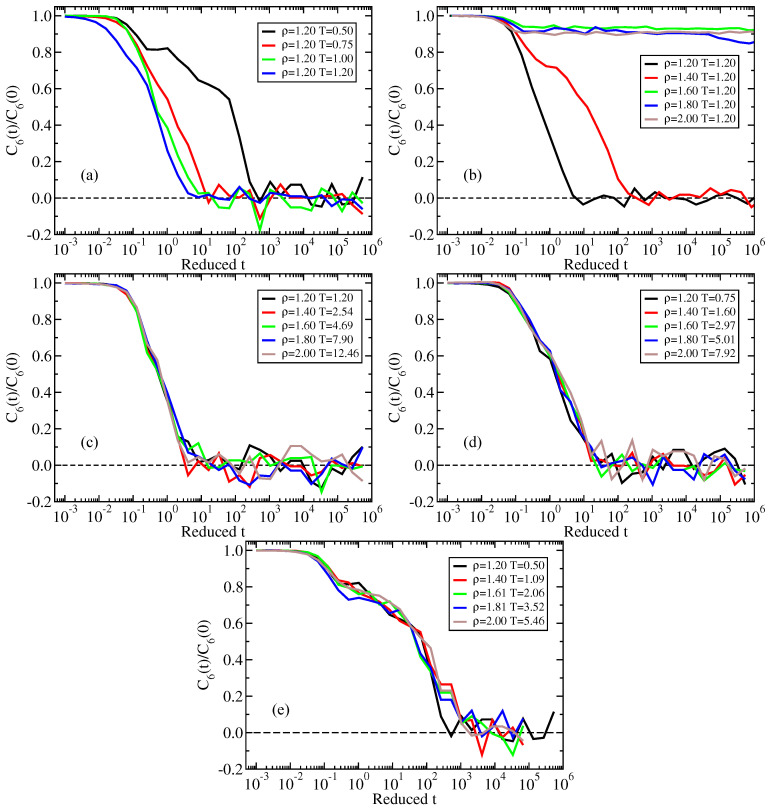
Normalized time-autocorrelation function of ${\bar{Q}}_{6}\left(t\right)$ plotted as a function of the reduced time. (**a**) Results along the ρ=1.20 isochore. (**b**) Results along the T=1.20 isotherm. (**c**) Results along the reference-state-point (ρ,T)=(1.20,1.20) isomorph. (**d**) Results along the reference-state-point (ρ,T)=(1.20,0.75) isomorph. (**e**) Results along the reference-state-point (ρ,T)=(1.20,0.50) isomorph. There is good isomorph invariance.

Figure 8a shows the normalized bond-orientational time-autocorrelation function of reduced time along an isochore (Figure 8a), an isotherm (Figure 8b), and three isomorphs (Figure 8c–e). We find good isomorph invariance.

#### 2.2.3. ${\mathrm{NiY}}_{2}$ Mixture

The third simulated binary LJ mixture involves parameters that have been determined by fitting to experimental data of the ${\mathrm{NiY}}_{2}$ (1:2 Nickel-Yttrium) metallic glass [57,58]. A particles mimic Yttrium atoms and B particles mimic Nickel atoms. The LJ interaction parameters are: $\sigma_{AA}=1.0$, $\sigma_{AB}=0.7727$, $\sigma_{BB}=0.6957$, and $\varepsilon_{AA}=\varepsilon_{AB}=\varepsilon_{BB}=1.0$. The mass of particle B is equal to that of particle A. A shifted-force cutoff was used at $r_{cut}=2.5\;\sigma$ with the relevant σ for each of the three interactions [59]. A system of 3200 A particles and 1600 B particles was studied using standard Nosé-Hoover NVT simulations with time step $0.001\rho^{-1/3}T^{-1/2}$ and a thermostat relaxation time of 0.2. Most systems were equilibrated for 3 million time steps before a data collection run of 10 million steps, but for the T=0.50 and T=0.55 state points on the isochore, equilibration and production runs were 300 and 50 million time steps, respectively. The isomorph reference state point was (ρ,T)=(1.30,1.00) (in A particle units). Isomorphic state points were generated from the reference state point using Equation (5). Voronoi structures were calculated using the Voro++ library [45].

**Figure 9:**
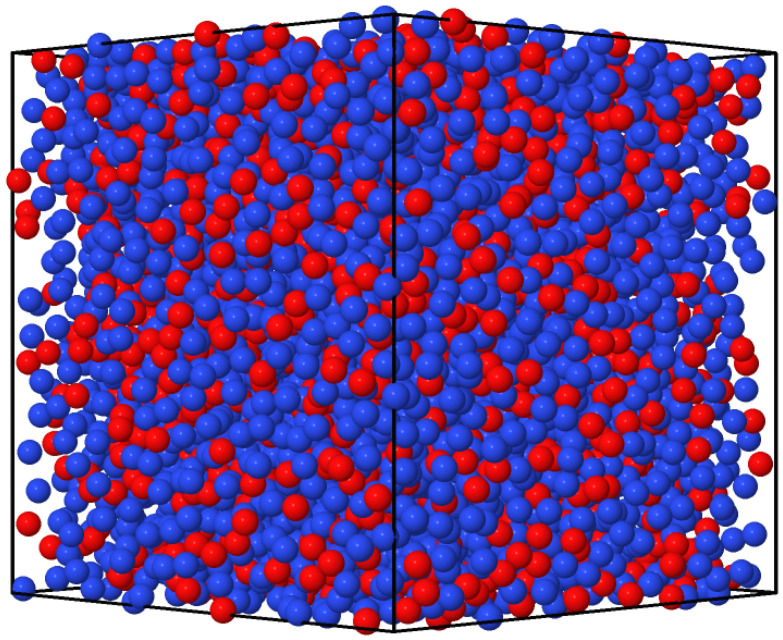
Snapshot of the ${\mathrm{NiY}}_{2}$ binary LJ system at (ρ,T)=(1.3,1.0), the reference state point for the isomorph studied. The A particles representing the Yttrium atoms are blue and the B particles representing the Nickel atoms are red. We see that the system is homogeneous.

Figure 9 shows a snapshot of the system equilibrated at the reference state point of the isomorph, demonstrating a homogeneous liquid state.

**Figure 10:**
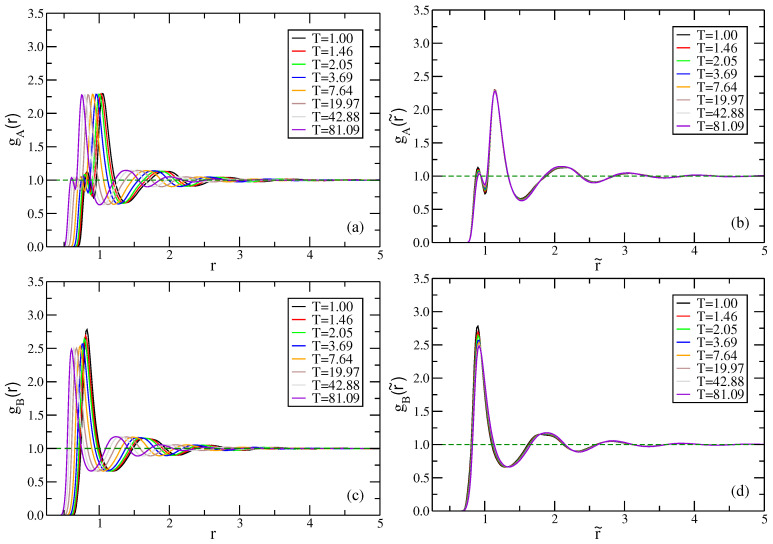
(**a**–**d**): RDF of central particles *A* or *B* counting all surrounding particles independent of their identity, monitored along the isomorph, plotted in both LJ units (**left**) and reduced units (**right**). There is good isomorph invariance of the reduced RDFs, although the first peak of the B particle RDF is visibly not isomorph invariant.

Isomorphs of the ${\mathrm{NiY}}_{2}$ model have not been studied before, so we first discuss the variation of different radial distribution functions (RDF) and the mean-square displacements (MSD) along the isomorph. The RDF data for the AA, AB, and BB distributions (data not shown) show good isomorph invariance in reduced units, except for the first peak of the AB RDF that decreases significantly with increasing temperature. We also investigated the less standard “total” A and B RDF functions defined by counting all surrounding particles (Figure 10). These RDFs focus on the surroundings of a given particle, ignoring which kind particles are involved. There is good invariance of the reduced total RDFs, with the exception of the first peak of the B particle RDF.

**Figure 11:**
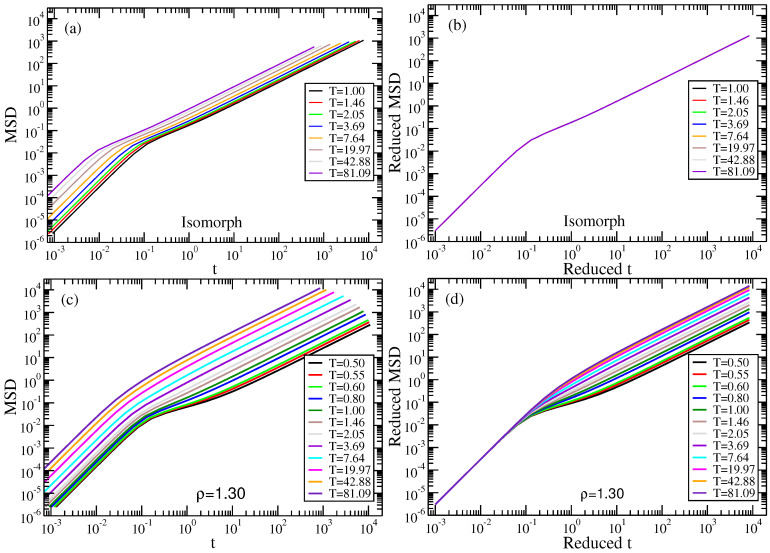
A particle (Y atom) MSD of the ${\mathrm{NiY}}_{2}$ binary LJ mixture along the isomorph in LJ and reduced units (**a**,**b**), and similarly along the ρ=1.30 isochore (**c**,**d**). Only along the isomorph is the MSD invariant in reduced units.

Figure 11 gives MSD data along the isomorph and an isochore. The upper figures give the isomorph MSD as a function of time in LJ units (left) and reduced units (right). The lower figures give the same for the ρ=1.30 isochore. We again remind that the short-time ballistic-region collapse in reduced units seen for both the isomorph and the isochore follows from the definition of reduced units (compare Figure 1, Figure 3, and Figure 6). The reduced-unit A particle MSD is isomorph invariant, while the three other MSD versions are not. We take Figure 10 and Figure 11 as a confirmation that a proper isomorph has been identified.

**Figure 12:**
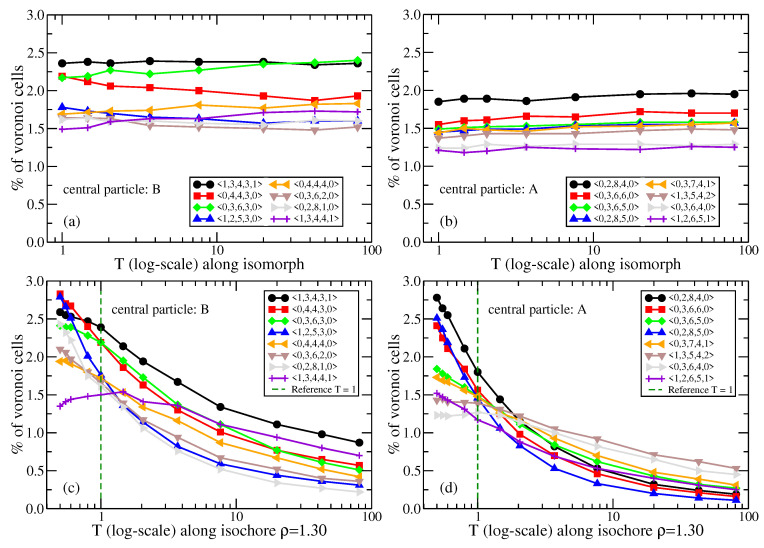
Temperature dependence of the occurrence of eight of the most abundant Voronoi structures at the reference state point (ρ,T)=(1.30,1.00), plotted for the ${\mathrm{NiY}}_{2}$ binary mixture as follows: along the isomorph as a function of the logarithm of the temperature (**a**,**b**), along the ρ=1.3 isochore as a function of the logarithm of the temperature (**c**,**d**). In (**a**,**c**) the central particle is of type B, in (**b**,**d**) it is an A particle. The Voronoi structures are isomorph invariant to a good approximation, but vary significantly along the isochore.

Having validated the standard structure and dynamics isomorph invariants for the ${\mathrm{NiY}}_{2}$ mixture, we proceed in Figure 12 to investigate how the higher-order structures vary along the isomorph, quantified by the occurrence of eight of the most common Voronoi structures identified at the reference state point Figure 12a,b. We present in Figure 12c,d analogous results along an isochore. The higher-order structures are approximately isomorph invariant, but vary significantly along the isochore.

## 3. Summary

This paper has demonstrated good isomorph invariance of different measures of higher-order structure in four LJ systems. The measures studied are the occurrence of Voronoi structures, the number of Frank–Kasper bonds, the icosahedral local order, and the time-autocorrelation function of the bond-orientational order. Our findings confirm the isomorph-theory prediction that structure is approximately isomorph invariant in reduced units, thus demonstrating that this property is not limited to the RDF [18,20,22]. On this background, the poor isomorph invariance of the bicapped 11A structure of the KA system reported in Ref. [40] represents an interesting exception. With isomorph theory in mind, the finding that this particular structure is not isomorph invariant indicates that its prevalence may not be important for the dynamics of the KA system, which *is* isomorph invariant. This argument illustrates that a breakdown of an isomorph-theory prediction can provide important information about a system.

## Data Availability

The data of this study are available upon request.

## Appendix A. Isomorph State-Point Data

We here give density ρ, temperature *T*, virial potential-energy correlation coefficient *R*, and density-scaling exponent γ of the state points studied on isomorphs.

**Table A1:** Selected LJ state points of isomorph 1 (top) and isomorph 2 (bottom) (Figure 1 and Figure 2).

ρ	*T*	*R*	γ
1.000	2.000	0.994	5.021
1.050	2.545	0.995	4.881
1.100	3.186	0.997	4.768
1.150	3.930	0.997	4.676
1.200	4.788	0.998	4.608
1.250	5.772	0.998	4.544
0.850	0.600	0.956	5.895
0.900	0.832	0.979	5.564
0.950	1.116	0.987	5.293
1.000	1.458	0.993	5.114
1.050	1.864	0.995	4.957
1.100	2.340	0.996	4.827
1.150	2.894	0.997	4.733
1.200	3.534	0.998	4.653

**Table A2:** Selected Wahnström state points of the (ρ,T) = (0.85, 1.2) reference-state-point isomorph (top) and the (ρ,T) = (0.85, 2.0) reference-state-point isomorph (bottom) (Figure 3, Figure 4 and Figure 5).

ρ	*T*	*R*	γ
0.75	0.646	0.982	5.050
0.80	0.893	0.990	4.911
0.85	1.200	0.994	4.785
0.90	1.572	0.996	4.681
0.95	2.018	0.997	4.596
1.00	2.549	0.998	4.525
1.50	14.738	0.999	4.205
2.00	48.610	0.999	4.110
0.65	0.493	0.936	5.600
0.70	0.748	0.977	5.364
0.75	1.076	0.988	5.106
0.80	1.489	0.993	4.912
0.85	2.000	0.995	4.772
0.90	2.620	0.997	4.659
0.95	3.364	0.998	4.573
1.00	4.248	0.998	4.505
1.50	24.564	0.999	4.197
2.00	81.016	0.999	4.105
2.50	201.615	0.999	4.066

**Table A3:** Selected KA state points of the four isomorphs (Figure 6, Figure 7 and Figure 8).

ρ	*T*	*R*	γ
1.200	0.500	0.939	5.158
1.403	1.091	0.983	4.784
1.607	2.058	0.993	4.568
1.810	3.520	0.997	4.424
2.001	5.461	0.998	4.339
1.200	0.750	0.958	5.149
1.400	1.601	0.988	4.774
1.600	2.966	0.995	4.552
1.800	5.009	0.997	4.415
2.000	7.916	0.999	4.324
1.200	1.000	0.968	5.111
1.400	2.126	0.990	4.743
1.600	3.929	0.996	4.530
1.800	6.625	0.998	4.399
2.000	10.459	0.999	4.311
1.200	1.200	0.973	5.081
1.400	2.542	0.992	4.721
1.600	4.689	0.996	4.514
1.800	7.897	0.998	4.387
2.000	12.459	0.999	4.303

**Table A4:** Selected ${\mathrm{NiY}}_{2}$ mixture state points of the isomorph (Figure 9, Figure 10, Figure 11 and Figure 12).

ρ	*T*	*R*	γ
1.30	1.000	0.959	5.270
1.40	1.461	0.980	5.083
1.50	2.050	0.988	4.889
1.70	3.689	0.995	4.645
2.00	7.640	0.998	4.430
2.50	19.965	0.999	4.253
3.00	42.877	0.999	4.173
3.50	81.086	0.999	4.123
